# Supplementary material for: GLYCO-BUILD: an enzymatic pipeline for the synthesis of peptides carrying eukaryotic N-glycans
Source: Nat Commun. 2025 Nov 29;17:369. doi: 10.1038/s41467-025-67055-2 (PMC12796225; doi:10.1038/s41467-025-67055-2)

SUPPLEMENTARY INFORMATION

GLYCO-BUILD: An enzymatic pipeline for the synthesis of peptides carrying eukaryotic *N*-glycans

Lorenzo Rossi[1,#], J. Andrew N. Alexander[1,#], Ana S. Ramírez[1,2,#] and Kaspar P. Locher[1*]

[1]Institute of Molecular Biology and Biophysics, Eidgenössische Technische Hochschule (ETH), Zürich, Switzerland.

[#]These authors contributed equally.

[2]Current address: Complex Carbohydrate Research Center, University of Georgia, Athens, GA, USA

*To whom correspondence should be addressed. Email: locher@mol.biol.ethz.ch

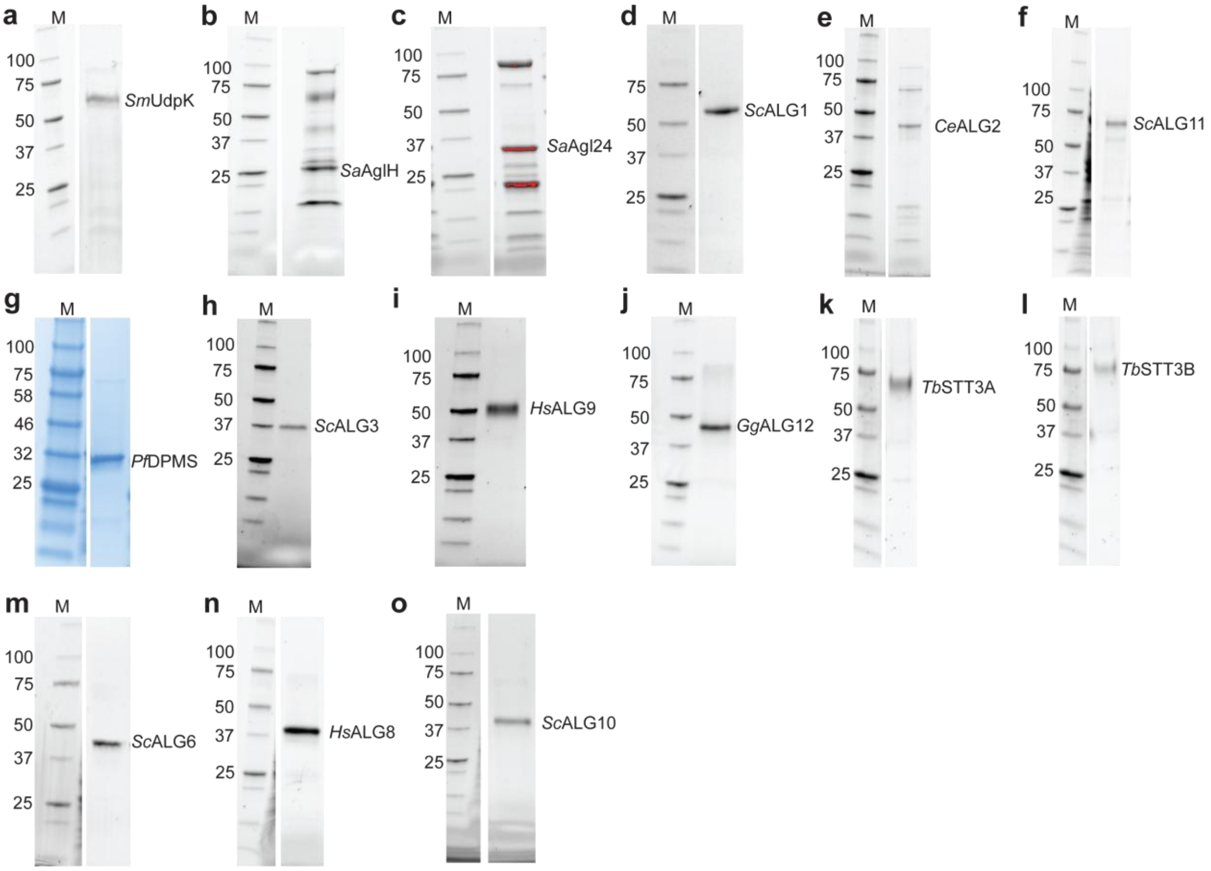

Supplementary Figure 1: SDS-PAGE analysis of purified enzymes used in GLYCO-BUILD. a, *Streptococcus mutans* UdpK, **b,** *Sulfolobus acidocaldarius* AglH, **c,** *Sulfolobus acidocaldarius* Agl24, **d,** *Saccharomyces cerevisiae* ALG1, **e,** *Caenorhabditis elegans* ALG2, **f,** *Saccharomyces cerevisiae* ALG11, **g,** *Pyrococcus furiosus* DPMS, **h,** *Saccharomyces cerevisiae* ALG3, **i,** *Homo sapiens* ALG9, **j,** *Gallus gallus* ALG12, **k,** *Trypanosoma brucei* STT3A, **l,** *Trypanosoma brucei* STT3B, **m,** *Saccharomyces cerevisiae* ALG6, **n,** *Homo sapiens* ALG8 and **o,** *Saccharomyces cerevisiae* ALG10. The molecular masses of the protein markers are shown in kDa.

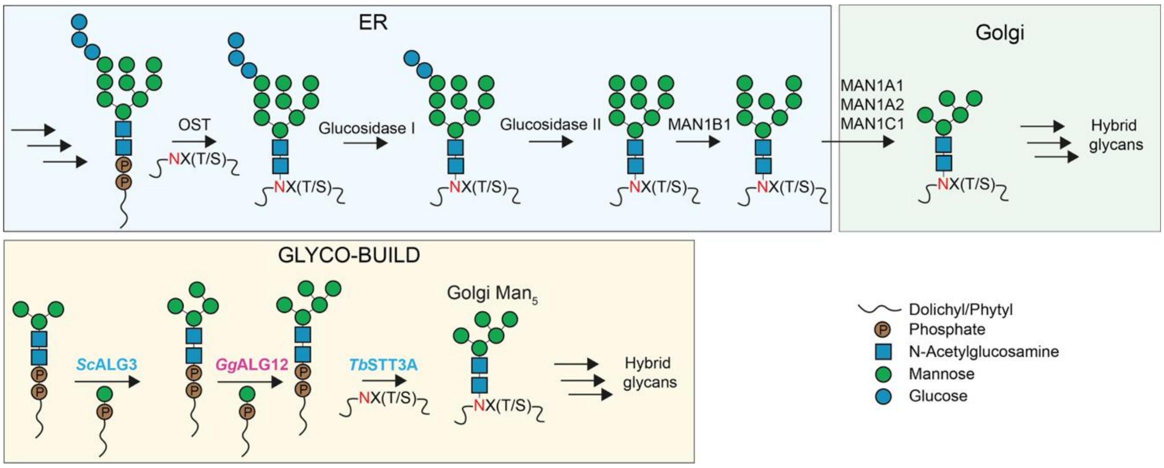

Supplementary Figure 2: Comparison of hybrid glycan synthesis in eukaryotes and using the GLYCO-BUILD pipeline. Steps taking place in the ER and Golgi are shown on a blue and green background, respectively. The GLYCO-BUILD *in vitro* synthesis pathway is shown on a tan background. Glycoforms are indicated using the symbol nomenclature for glycans (SNFG).

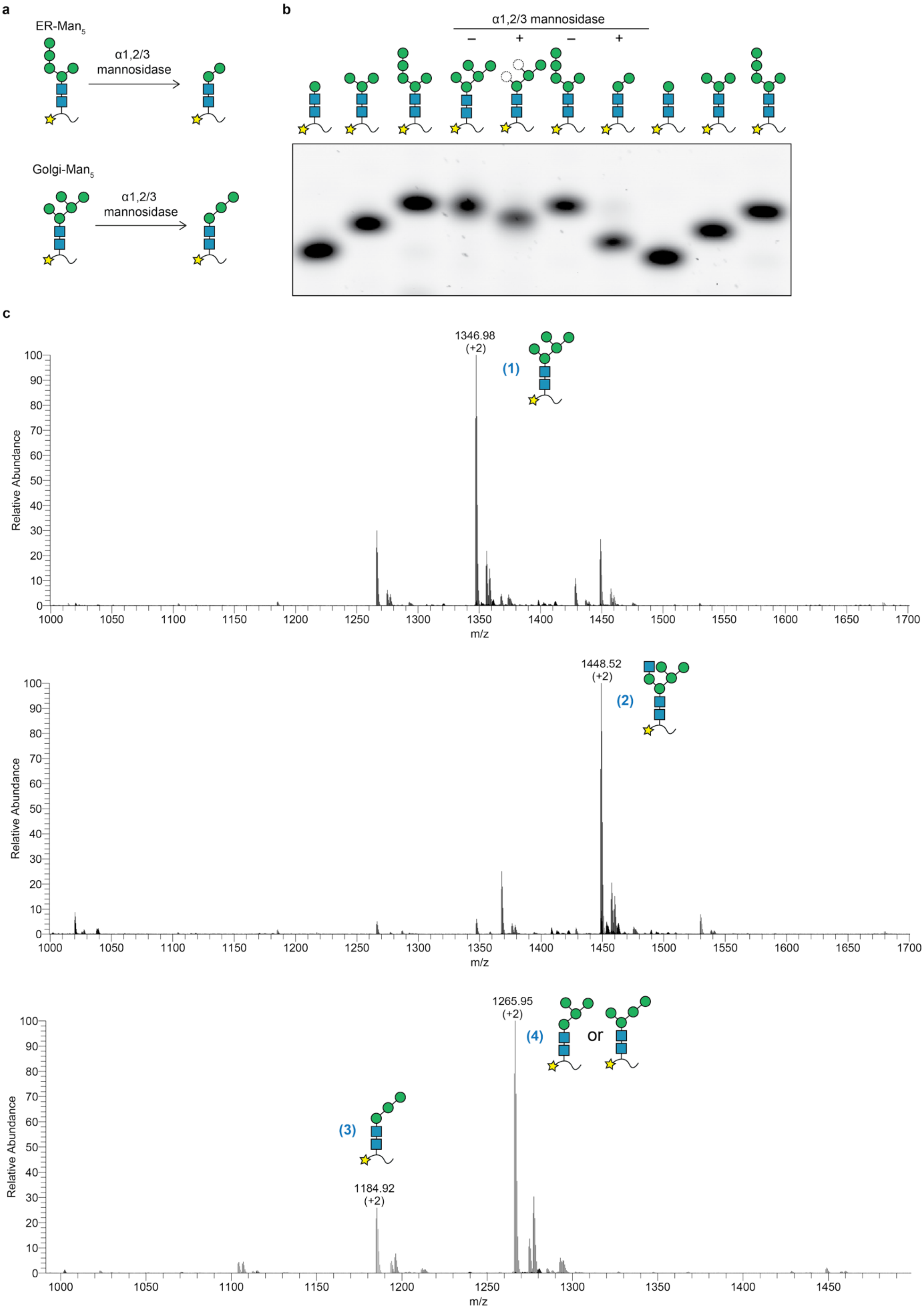

Supplementary Figure 3: Validation of ER- and Golgi-Man$_5$ glycan structures. a, Schematics of ER- and Golgi-Man$_5$ glycan digestion with an α1,2/3 mannosidase. **b,** Tricine gel electrophoresis of ER- and Golgi-Man$_5$ glycopeptides with and without the addition of α1,2/3 mannosidase. GlcNAc$_2$Man$_1$, GlcNAc$_2$Man$_3$ and GlcNAc$_2$Man$_5$ glycopeptides are also shown on the gel as references. **c,** LC-MS

analysis of selected glycopeptides. (1) HRMS (m/z): $[M+2H]^{2+}$ calcd. for $C_{114}H_{155}N_{15}O_{60}^{2+}$, 1346.98; found, 1346.98. (2) HRMS (m/z): $[M+2H]^{2+}$ calcd. for $C_{122}H_{166}N_{16}O_{65}^{2+}$, 1448.52; found, 1448.52. (3) HRMS (m/z): $[M+2H]^{2+}$ calcd. for $C_{102}H_{135}N_{15}O_{50}^{2+}$, 1184.92; found, 1184.92. (4) HRMS (m/z): $[M+2H]^{2+}$ calcd. for $C_{108}H_{145}N_{15}O_{55}^{2+}$, 1265.95; found, 1265.95. (1) and (2) represent the Golgi-Man_5 and Golgi-Man_5GlcNAc samples, respectively. (3) and (4) represent the main products from the α1,2/3 mannosidase digestion of Golgi-Man_5. Glycoforms are indicated using the symbol nomenclature for glycans (SNFG).

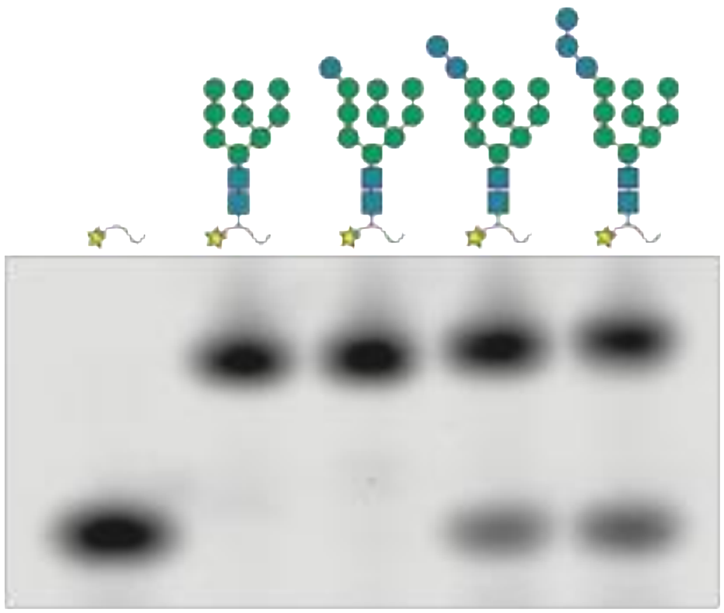

Supplementary Figure 4: Electrophoretic analysis of glucosylated *N*-glycans synthesized and transferred using GLYCO-BUILD. Assembled glycans were transferred from phytyl-PP-linked oligosaccharides to the peptide 5-carboxyfluorescein-GSLANYTK using *Tb*STT3B and separated on tricine gel. To validate the small shifts visible between the lanes, selected glycans were analyzed by mass spectrometry (Fig. 3a,b of the main text). The glycoforms loaded are indicated above the lanes using the symbol nomenclature for glycans (SNFG), the peptide is depicted by a black line, and the fluorophore is depicted with a yellow star.

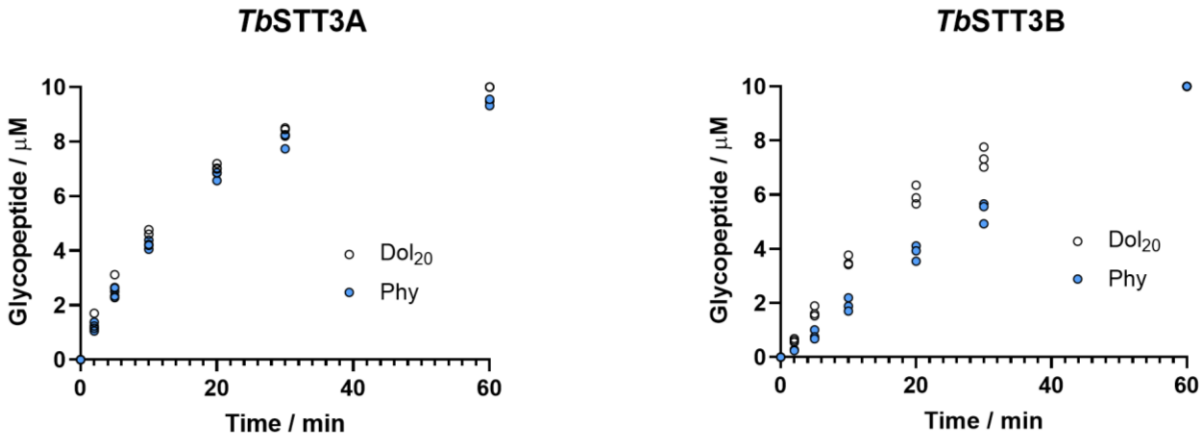

Supplementary Figure 5: Effect of lipid tail composition for *in vitro* glycosylation reactions catalyzed by single-subunit OST enzymes. Left, time course of 5-carboxyfluorescein-GSDANYTYTQ glycosylation by *Tb*STT3A using Dol_{20}-PP-$GlcNAc_2Man_5$ or Phy-PP-$GlcNAc_2Man_5$ as donor substrates. Right, time course of 5-carboxyfluorescein-GSLANYTK glycosylation by *Tb*STT3B using Dol_{20}-PP-$GlcNAc_2Man_9$ or Phy-PP-$GlcNAc_2Man_9$ as donor substrates. Data (n=3 per condition) are presented as individual data points.

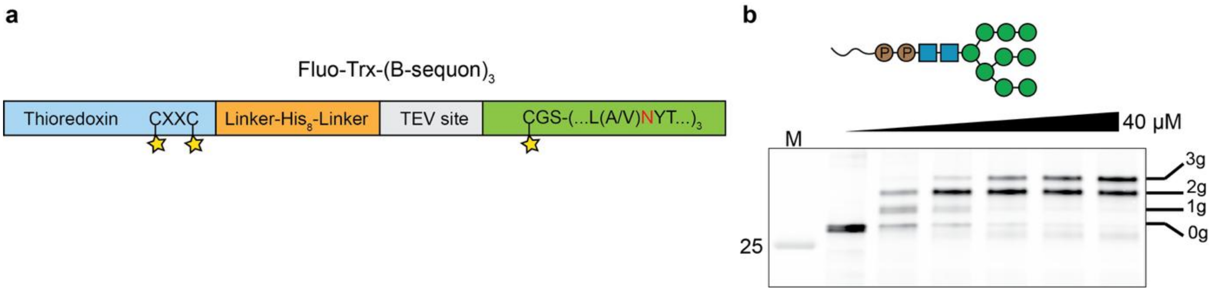

Supplementary Figure 6: SDS-PAGE analysis of synthesized glycoprotein, containing three glycosylation sequons. a, Construct design of a thioredoxin with three C-terminal B-sequons. The glycosylation sequons are shown with the acceptor asparagine colored in red. Cysteine residues are indicated bound to fluorescein, represented by a yellow star. **b,** *Tb*STT3B catalyzed glycoprotein synthesis with Phy-PP-GlcNAc$_2$Man$_9$ glycan. Fluorescently labelled Trx-(*Tb*STT3B-sequon)$_3$ was incubated with increasing amounts of LLO (0 µM, 5 µM, 10 µM, 20 µM, 30 µM, 40 µM) and *Tb*STT3B overnight before the products were separated on an SDS-PAGE gel. Bands are labelled with 0g, 1g, 2g, or 3g to indicate 0, 1, 2 or 3 attached glycans, respectively. The molecular weight of the fluorescent protein standard is indicated in kDa. Glycoforms used for glycosylation are depicted using the symbol nomenclature for glycans (SNFG).

Peptide	Glycan	Observed *m/z* $[M+2H]^{2+}$	Calculated *m/z* $[M+2H]^{2+}$
5-TAMRA-YA**N**ATS-NH_2	$GlcNAc_2$	722.30	722.30
	$GlcNAc_2Man_3$	965.38	965.38
	$GlcNAc_2Man_5$	1127.44	1127.43
	$GlcNAc_2Man_9$	1451.54	1451.54
	$GlcNAc_2Man_9Glc_3$	1694.63	1694.62

Supplementary Table 1: LC-MS analysis of the glycopeptides generated by *Sc*OST-mediated transfer of phytyl-pyrophosphate-oligosaccharides assembled using GLYCO-BUILD enzymes. The peptide sequence is represented using the single-letter code for amino acids (YANATS). Glycans are linked to the asparagine (N) residue highlighted in bold. 5-TAMRA: 5-carboxytetramethylrhodamine; GlcNAc: N-acetylglucosamine; Man: mannose; Glc: glucose. MS spectra are displayed in Figure 3b of the main text.

Uncropped gels for Supplementary Figure 1a-o:

*Sm*UdpK:

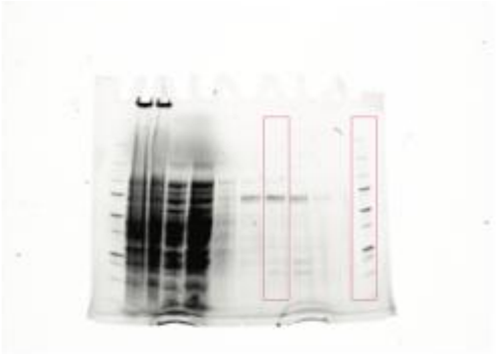

*Sa*AglH:

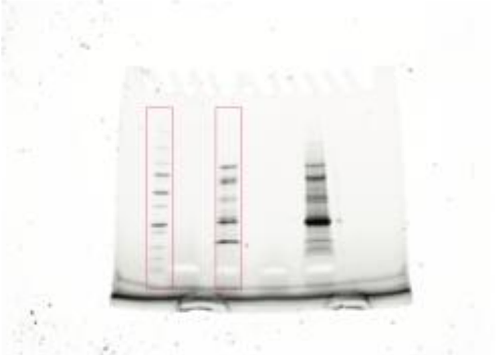

*Sa*Agl24:

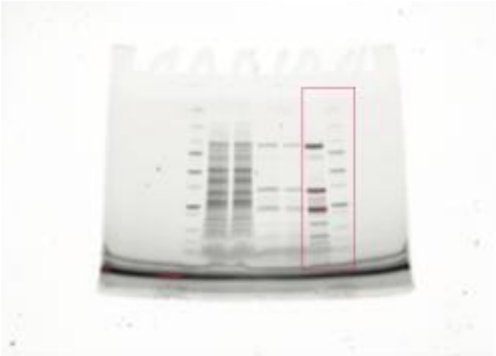

*Sc*ALG1:

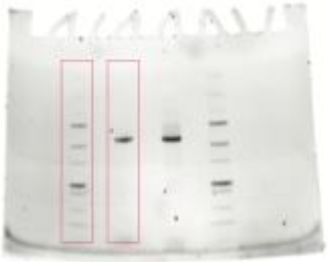

*Ce*ALG2:

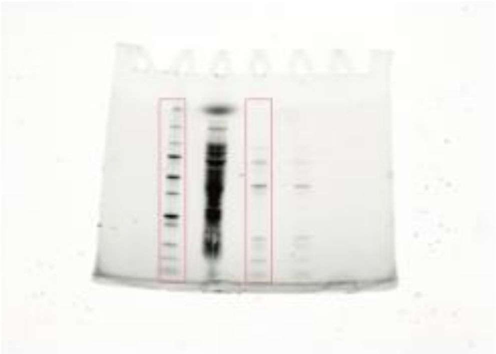

*Sc*ALG11:

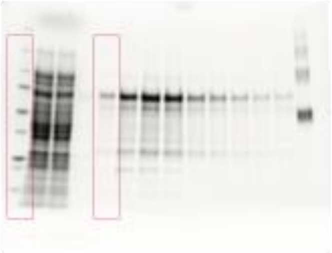

*Pf*DPMS:

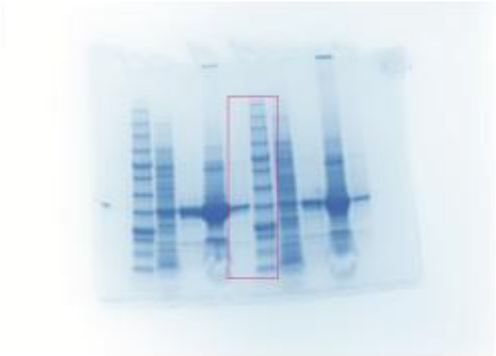

*Sc*ALG3:

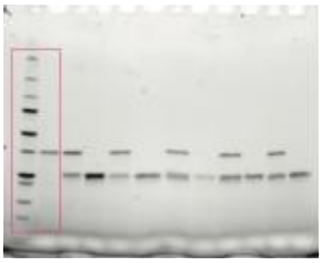

*Hs*ALG9:

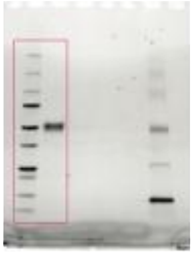

*Gg*ALG12:

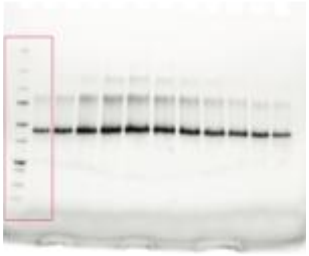

*Tb*STT3A and *Tb*STT3B:

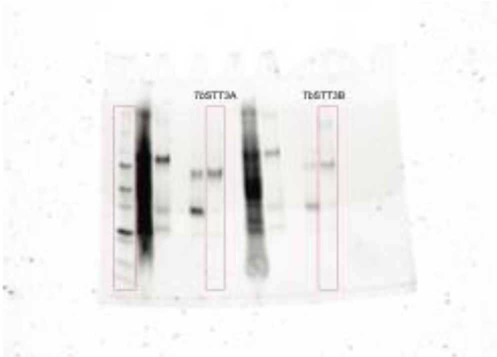

ScSALG6:

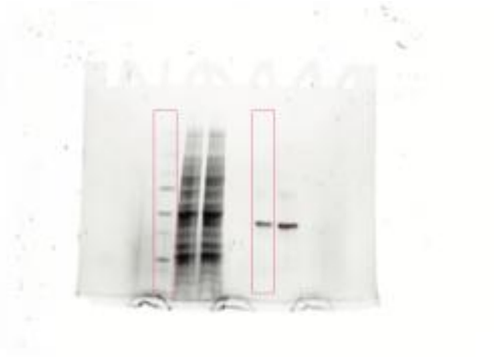

*Hs*ALG8:

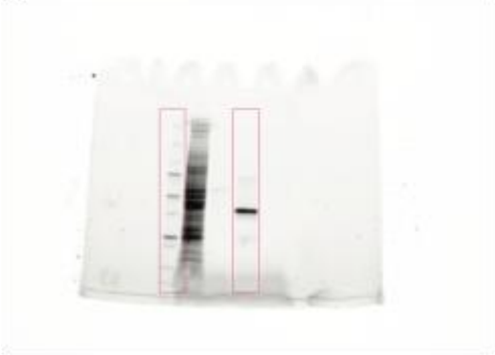

*Sc*ALG10:

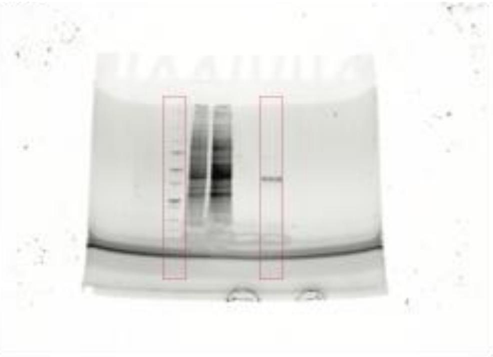

Uncropped gel for Supplementary Figure 3b:

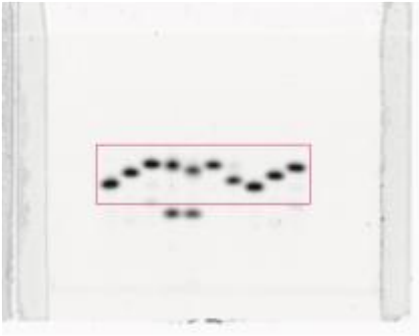

Uncropped gel for Supplementary Figure 4:

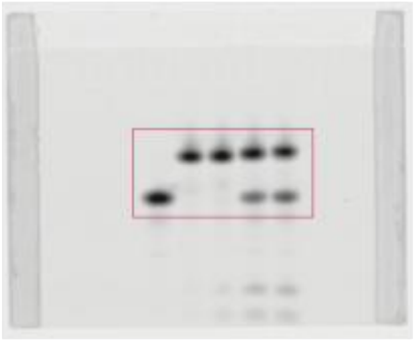

Uncropped gel for Supplementary Figure 6b:

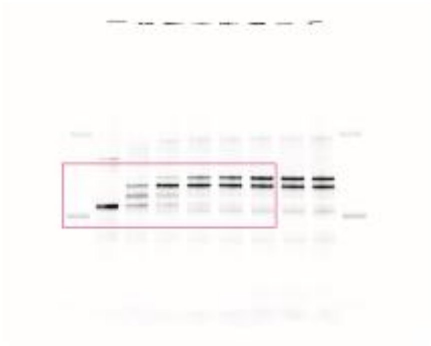

Supplement: Supplementary file 1 — Supplementary Information [file 41467_2025_67055_MOESM1_ESM.pdf]
