# Supplementary material for: GLYCO-BUILD: an enzymatic pipeline for the synthesis of peptides carrying eukaryotic N-glycans
Source: Nat Commun. 2025 Nov 29;17:369. doi: 10.1038/s41467-025-67055-2 (PMC12796225; doi:10.1038/s41467-025-67055-2)
Supplement: Supplementary file 2 — Reporting Summary [file 41467_2025_67055_MOESM2_ESM.pdf]

nature portfolio

Corresponding author(s): Kaspar P. Locher

Last updated by author(s): Nov 18, 2025

Reporting Summary

Nature Portfolio wishes to improve the reproducibility of the work that we publish. This form provides structure for consistency and transparency in reporting. For further information on Nature Portfolio policies, see our Editorial Policies and the Editorial Policy Checklist.

Statistics

For all statistical analyses, confirm that the following items are present in the figure legend, table legend, main text, or Methods section.

n/a	Confirmed	
☐	☒	The exact sample size (n) for each experimental group/condition, given as a discrete number and unit of measurement
☐	☒	A statement on whether measurements were taken from distinct samples or whether the same sample was measured repeatedly
☒	☐	The statistical test(s) used AND whether they are one- or two-sided<br>*Only common tests should be described solely by name; describe more complex techniques in the Methods section.*
☒	☐	A description of all covariates tested
☒	☐	A description of any assumptions or corrections, such as tests of normality and adjustment for multiple comparisons
☐	☒	A full description of the statistical parameters including central tendency (e.g. means) or other basic estimates (e.g. regression coefficient) AND variation (e.g. standard deviation) or associated estimates of uncertainty (e.g. confidence intervals)
☒	☐	For null hypothesis testing, the test statistic (e.g. F, t, r) with confidence intervals, effect sizes, degrees of freedom and P value noted<br>*Give P values as exact values whenever suitable.*
☒	☐	For Bayesian analysis, information on the choice of priors and Markov chain Monte Carlo settings
☒	☐	For hierarchical and complex designs, identification of the appropriate level for tests and full reporting of outcomes
☒	☐	Estimates of effect sizes (e.g. Cohen's d, Pearson's r), indicating how they were calculated

Our web collection on statistics for biologists contains articles on many of the points above.

Software and code

Policy information about availability of computer code

Data collection	Azure Sapphire
Data analysis	Data analysis and visualization: UCSF ChimeraX 1.6.1, GraphPad Prism 10, Clustal Omega 1.2.2, ESPript 3.0, ImageJ, Chromeleon, Xcalibur

For manuscripts utilizing custom algorithms or software that are central to the research but not yet described in published literature, software must be made available to editors and reviewers. We strongly encourage code deposition in a community repository (e.g. GitHub). See the Nature Portfolio guidelines for submitting code & software for further information.

Data

Policy information about availability of data

All manuscripts must include a data availability statement. This statement should provide the following information, where applicable:

- Accession codes, unique identifiers, or web links for publicly available datasets
- A description of any restrictions on data availability
- For clinical datasets or third party data, please ensure that the statement adheres to our policy

Unless otherwise stated, all data supporting the results of this study can be found in the article, supplementary, and source data files. The mass spectrometry glycoproteomics data generated in this study have been deposited in the GlycoPOST database under accession code GPST000641 [http://doi.org/10.50821/GLYCOPOST-GPST000641] and to the ProteomeXchange Consortium via PRIDE partner repository with the identifier PXD070840 [https://proteomecentral.proteomexchange.org/cgi/GetDataset?ID=PXD070840]. The protein purification and activity data generated in this study are provided in the Supplementary Information. Source data are provided with this paper.

Research involving human participants, their data, or biological material

Policy information about studies with human participants or human data. See also policy information about sex, gender (identity/presentation), and sexual orientation and race, ethnicity and racism.

Reporting on sex and gender	not applicable
Reporting on race, ethnicity, or other socially relevant groupings	not applicable
Population characteristics	not applicable
Recruitment	not applicable
Ethics oversight	not applicable

Note that full information on the approval of the study protocol must also be provided in the manuscript.

Field-specific reporting

Please select the one below that is the best fit for your research. If you are not sure, read the appropriate sections before making your selection.

- [x] Life sciences
- [ ] Behavioural & social sciences
- [ ] Ecological, evolutionary & environmental sciences

For a reference copy of the document with all sections, see nature.com/documents/nr-reporting-summary-flat.pdf

Life sciences study design

All studies must disclose on these points even when the disclosure is negative.

Sample size	Kinetic studies of glycopeptides synthesis using TbSTT3A and TbSTT3B were performed with N=3 biological replicates per timepoint. Kinetic studies of Dol-linked vs Phy-linked LLOs usage by TbSTT3A and TbSTT3B were performed with N=3 biological replicates per timepoint. Expression and purification of proteins, and functional activity assays were performed a minimum of N=3 times. This number is accepted to evaluate reliability of the protocol and to calculate mean and standard deviation for enzymatic assays.
Data exclusions	No data was excluded from the analyses
Replication	Kinetic studies and glycopeptides/glycoproteins synthesis attempts were replicated and reproduced successfully multiple (>3) times. Same is true for protein expression and purification attempts. Viral peptide glycosylation was performed >4 times with similar results.
Randomization	not applicable protein expression, purification and enzymatic synthesis studies
Blinding	not applicable protein expression, purification and enzymatic synthesis studies

Reporting for specific materials, systems and methods

We require information from authors about some types of materials, experimental systems and methods used in many studies. Here, indicate whether each material, system or method listed is relevant to your study. If you are not sure if a list item applies to your research, read the appropriate section before selecting a response.

Materials & experimental systems

n/a	Involved in the study	
x		Antibodies
	x	Eukaryotic cell lines
x		Palaeontology and archaeology
x		Animals and other organisms
x		Clinical data
x		Dual use research of concern
x		Plants

Methods

n/a	Involved in the study	
x		ChIP-seq
x		Flow cytometry
x		MRI-based neuroimaging

Eukaryotic cell lines

Policy information about cell lines and Sex and Gender in Research

Cell line source(s)	Human Embrionic Kidney (HEK) suspension 293 c18 (ATCC CRL-10852); Spodoptera frugiperda Sf9 (ATCC CRL-1711); Saccharomyces cerevisiae BY4742 Δost6::Leu, OST4-1D4::KanX. The ATCC for the parental yeast strain BY4742 is ATCC

	201389.
Authentication	No authentication was performed
Mycoplasma contamination	No contamination tests were performed
Commonly misidentified lines (See ICLAC register)	Not applicable

Plants

Seed stocks	Not applicable
Novel plant genotypes	Not applicable
Authentication	Not applicable
